# Response of grassland ecosystem function to plant functional traits under different vegetation restoration models in areas of karst desertification

**DOI:** 10.3389/fpls.2023.1239190

**Published:** 2023-12-12

**Authors:** Shuzhen Song, Kangning Xiong, Yongkuan Chi

**Affiliations:** ^1^ School of Karst Science, Guizhou Normal University, Guiyang, China; ^2^ State Engineering Technology Institute for Karst Desertification Control, Guizhou Normal University, Guiyang, China

**Keywords:** plant diversity, functional trait, ecosystem function, ecological restoration, karst desertification

## Abstract

Plant functional traits serve as a bridge between plants, the environment, and ecosystem function, playing an important role in predicting the changes in ecosystem function that occur during ecological restoration. However, the response of grassland ecosystem function to plant functional traits in the context of ecological restoration in areas of karst desertification remains unclear. Therefore, in this study, we selected five plant functional traits [namely, plant height (H), specific leaf area (SLA), leaf dry matter content (LDMC), root length (RL), and root dry matter content (RDMC)], measured these along with community-weighted mean (CWM) and functional trait diversity, and combined these measures with 10 indexes related to ecosystem function in order to investigate the differences in plant functional traits and ecosystem function, as well as the relationship between plant functional traits and ecosystem functions, under four ecological restoration models [*Dactylis glomerata* (DG), *Lolium perenne* (LP), *Lolium perenne* + *Trifolium repens* (LT), and natural grassland (NG)]. We found that: 1) the Margalef index and Shannon–Wiener index were significantly lower for plant species in DG and LP than for those in NG (*P*<0.05), while the Simpson index was significantly higher in the former than in NG (*P*<0.05); 2) CWM_H_, CWM_LDMC_, and CWM_RDMC_ were significantly higher in DG, LP, and LT than in NG, while CWM_SLA_ was significantly lower in the former than in NG (*P*<0.05). The functional richness index (FRic) was significantly higher in DG and LP than in NG and LT, but the functional dispersion index (FDis) and Rao’s quadratic entropy index (RaoQ) were significantly lower in DG and LP than in NG and LT (*P*<0.05), and there was no significant difference between DG and LP, or between NG and LT (*P*>0.05); 3) ecosystem function, including ecosystem productivity, carbon storage, water conservation and soil conservation, was highest in LT and lowest in NG; and 4) CWM_LDMC_ (F=56.7, P=0.024), CWM_RL_ (F=28.7, P=0.024), and CWM_H_ (F=4.5, P=0.048) were the main factors affecting ecosystem function. The results showed that the mixed pasture of perennial ryegrass and white clover was most conductive to restoration of ecosystem function. This discovery has important implications for the establishment of vegetation, optimal utilization of resources, and the sustainable development of degraded karst ecosystems.

## Introduction

The findings of the Millennium Ecosystem Assessment report show that, globally, approximately 60% of the services provided by nature are currently being degraded or are in an unsustainable state. Furthermore, the report reveals that 78% of the benefits that humans derive from nature are rapidly declining (MEA, 2005). The sustainability of ecosystem services has become one of the most important issues for natural resource and environmental management in recent decades (Sharafatmandrad and Mashizi, 2020; Sardar et al., 2023). Biodiversity plays a crucial role in maintaining ecosystem services and human well-being, and the relationship between biodiversity and ecosystem functioning has become a hot topic in ecological research (Cardinale et al., 2012; Gamfeldt and Roger, 2017; Plas, 2019). Biodiversity is considered to be one of the fundamental indicators of ecosystem restoration (Liu M. et al., 2022). Restoring biodiversity and ecosystem function has become the primary objective in ecological restoration (Zirbel et al., 2017; Teng et al., 2023). Not only is an understanding of the relationship between biodiversity and ecosystem function helpful in better coping with the ecological consequences of biodiversity loss under a scenario of global climate change and increasing disturbance arising from human activities, but such an understanding also has practical implications for achieving sustainable management of ecosystems, ensuring the provision of ecosystem services, and realizing the goal of ecological restoration (Hector and Bagchi, 2007; Fry et al., 2018; Gonzalez et al., 2020; Miao et al., 2023). However, the outcomes of restoring biodiversity and ecosystem function are often unpredictable, and the question of how to link changes in community composition to ecosystem function is a major challenge facing current work in ecology and environmental management (Siwicka et al., 2020; Shabaan et al., 2022). Functional trait-based approaches provide a useful framework for studying plant resource acquisition, population survival strategies, and changes in ecosystem processes and functions during restoration (McCormack et al., 2017).

The term *functional traits* refers to morphological, physiological, and life history traits that indirectly influence plant fitness by affecting growth, reproduction, and survival at the individual level; these traits can be divided into response traits (traits related to the response of organisms to environmental factors, such as resources and disturbances) and effect traits (traits that determine the impact of organisms on ecosystem functions) (Violle et al., 2007). Functional traits are considered to be measurable characteristics that determine the interaction between plants and their environment (Diaz and Cabido, 2001), and have been used to study plant adaptive strategies and their effects on ecosystem function (Garnier et al., 2015). Plant functional traits, which serve as physiological and ecological indicators of plant uptake, use, and maintenance of resources, reflect plant adaptation to different environments and the physiological or evolutionary trade-offs between different functions within plants; these traits are bridges between plants, environments, and ecosystem functions, and play an important role in determining ecosystem functions (Liu et al., 2021). A growing body of research-based evidence shows that plant functional traits are closely related to ecosystem function and can, to some extent, indicate changes in ecosystem function (Faucon et al., 2015; Hobbie, 2015; Roumet et al., 2016). For example, traits related to plant structure and physiology, such as specific leaf area (SLA), stem dry matter content, and leaf nutrient concentration, determine the quality and quantity of litter produced, which in turn indirectly affects leaf carbon storage and decomposition rates (Kazakou et al., 2006). There are also related studies showing that leaf traits (e.g., SLA or carbon-to-nitrogen (C:N) ratio) and root traits related to nutrient access (e.g., diameter, nutrient content, branching intensity) may exert different effects on resource access, carbon storage, and pathogen communities in the soil (Albert et al., 2012; McCormack et al., 2015). However, since there are numerous plant species in nature and various types and intensities of climate change and disturbances arising from human activity, it has become crucial to determine how to utilize plant functional traits to accurately reflect and predict changes in plant communities and ecosystem functions in the process of ecological restoration (Lei et al., 2016).

Two hypotheses can explain the relationship between plant functional traits and ecosystem function (Cadotte, 2017). One is the “mass-ratio hypothesis”, which states that the relative biomass of dominant species in the plant community and their specific traits are the dominating factors in the dynamic changes that occur in ecosystem processes (Grime, 1998); this is often characterized using the community-weighted mean (CWM) (Garnier et al., 2004; Vile et al., 2006; Díaz et al., 2007) for measures such as primary productivity (Finegan et al., 2015; Duffy et al., 2017) and soil carbon storage (Cong et al., 2014; Lange et al., 2015). The other is the “niche differentiation hypothesis”, which suggests that trait differences between species maximize the diversity of resource-use strategies, enhance ecosystem processes by reducing niche overlap, and subsequently influence ecosystem functions, which are considered to be an important component of biodiversity (Petchey and Gaston, 2002; Díaz et al., 2007). Measures of the diversity of plant functional traits, including the functional richness index, functional evenness index, and functional divergence index, are typically used to assess this hypothesis (Tilman et al., 1997). For example, Qi et al. (2022) found that species diversity, functional diversity, and biodiversity in phylogenetic space were generally positively related to productivity in their examation of the relationship between biomass and species diversity, functional diversity, and phylogenetic diversity of each community in the grasslands of the Qinghai-Tibet Plateau. Zhang et al. (2023) investigated the contribution of intraspecific variability to ecosystem function and found that a community with high interspecific variation in plant height and individuals with large leaf area could exhibit improved productivity through niche complementarity and dominance effects, respectively. Because functional diversity can better quantify the trait differences that define species interactions, is more sensitive to environmental stress or disturbance, and is more indicative of changes in ecosystem function, an increasing number of studies have used functional diversity measures to examine the relationship between plant functional traits and ecosystem function (Flynn et al., 2011; Chollet et al., 2014; Gross et al., 2017; Rosenfield and Müller, 2020). However, the mass ratio hypothesis and the niche differentiation hypothesis are not mutually exclusive; they jointly explain the construction of plant communities and ecosystem functions or processes, and have different relative importance in explaining ecosystem functions under different environmental conditions and vegetation types (Schleuter et al., 2010; Mouillot et al., 2013; Kraft et al., 2015; Jiang M. et al., 2022). It is therefore crucial to screen for plant functional traits that are associated with specific ecosystem processes, taking into account regional differences and ecosystem variation.

The karst ecosystem is a significant component of the terrestrial ecosystem, covering approximately 15% of the world’s land area (Yuan, 1991). Among these regions, the South China Karst, with Guizhou as its center, is one of the three major areas of concentrated karst distribution in the world, and it is also the main area of karst ecosystem in China. Due to the fragility of the karst ecosystem itself, following long-term, unsustainable levels of development and use by humans, the ecosystem function is damaged and a rocky, desertified landscape appears (Xiong et al., 2002). The degradation and alteration of the ecological environment due to karst desertification result in decreased stability, weakened resistance to disturbances, and reduced biodiversity in the karst ecosystem. Consequently, the sustainable development of this region has attracted much attention from scholars (Xiong et al., 2012; Canedoli et al., 2021; Yang et al., 2021; Gu et al., 2022). In order to control karst desertification, a large number of ecological restoration projects have been carried out in the karst areas of southern China. Ecological restoration is the core task of karst desertification control, and its goal is to restore biodiversity and ecosystem functions (Benayas et al., 2009; Xiong et al., 2022). Realizing the sustainable development of the karst ecological environment is an important issue for current karst desertification control (He et al., 2019; Wang K. et al., 2020; Xiao and Xiong, 2022). The use of grassland is a pioneer strategy in terms of plant community ecosystems for ecological restoration, and “grain for green” and the establishment of artificial grassland are important components of the project to restore degraded ecosystems; these approaches play an irreplaceable role in the ecological restoration process (Chi et al., 2020). The results of ecological restoration and control over many years have shown that grasslands involved in rocky desertification control have a significant positive effect in terms of the restoration of degraded soil, biodiversity, and ecosystem function (Song et al., 2022). Therefore, a comprehensive and in-depth study on the response of grassland ecosystem function to plant functional traits under different restoration models in karst desertification control areas can provide a better understanding of the degradation and restoration processes of the karst ecosystem. Additionally, such work can provide a theoretical basis for the practice of ecological restoration.

So far, although scientists have carried out extensive research on the functional characteristics of karst plants in the context of ecological restoration, they have mainly focused on adaptive strategies. For example, Zhang S. et al. (2020) conducted a statistical analysis of the relationships among leaf functional traits, plant characteristics, and environmental factors in order to explore the ecological strategies and driving factors of dominant plants in different succession stages of the ecosystem under karst desertification. Liu L. et al. (2022) comprehensively investigated four forests (three natural secondary forests and one artificial forest) in a trough-valley karst watershed in northern Guizhou Province, southwest China, to examine the community-level adaptation strategies of karst forests. Zhou et al. (2022) studied the differences in species composition and functional characteristics between dolomite and limestone karst natural forests to clarify the adaptability of vegetation to desertified karst environments. However, there are fewer studies on the relationship between plant functional traits and ecosystem function, and research on the response of grassland ecosystem function to plant functional traits in the context of ecological restoration in areas of karst desertification is also at an exploratory stage. Therefore, there is an urgent need to explore the response mechanism of grassland ecosystem function to plant functional traits in the context of ecological restoration in areas of karst desertification. On this basis, the objectives of this study were: (1) to investigate the differences in plant functional traits and ecosystem functions between natural and artificially restored grassland ecosystems; and (2) to evaluate the relationship between plant functional traits and grassland ecosystem function. We hypothesized that CWM and functional trait diversity in plant functional traits in grassland ecosystems under different vegetation restoration models would be found to contribute equally to changes in grassland ecosystem function. To address this hypothesis, we selected five indicators of plant functional traits, namely plant height (H), specific leaf area (SLA), leaf dry matter content (LDMC), root length (RL), and root dry matter content (RDMC). We assessed CWM and functional trait diversity, and combined these measures with 10 ecosystem function-related indicators to comparatively analyze the response of grassland ecosystem function to plant functional traits under four ecological restoration models [*Dactylis glomerata* (DG) grassland, *Lolium perenne* (LP) grassland, *Lolium perenne* + *Trifolium repens* (LT) grassland, and natural grassland (NG)] employed in areas of karst desertification, with the aim of providing a scientific basis for vegetation construction, resource maximization, and sustainable development of degraded karst ecosystems.

## Materials and methods

### Study area

The study area is located in Salaxi Town and Yejiao Town, Qixingguan District, Bijie City, Guizhou Province, China (105°02′01′′–105°08′09′′E, 27°11′36′′–27°16′51′′N). The area of the region is 86.27 km^2^, and the area of rocky desertification is 55.931 km^2^, accounting for 64.93% of the total area. This area is a typical karst plateau mountain area with light-to-moderate rocky desertification. The study area has a subtropical humid monsoon climate, which is warm and humid in the summer and autumn, and cold and dry in the winter and spring. The average annual temperature is 12°C, the average annual rainfall is 984.40 mm, and rainfall is concentrated in the period from June to September. With a maximum elevation of 2,200 m and a minimum elevation of 1,495m, the terrain in the area varies greatly. The surface is fragmented and there are many peaks and depressions. The soil is mainly zonal yellow soil. The primary vegetation in the area has essentially been destroyed and the secondary vegetation now dominates; this includes *Cyclobalanopsis glauca*, *Pyracantha fortuneana*, *Pinus massoniana*, *Rhododendron simsii*, *Juglans regia*, *Rosa roxburghii*, *Artemisia lavandulaefolia*, *Chenopodium glaucum*, *Clinopodium chinense*, *Plantago asiatica*, *Stellaria media*, *Digitaria sanguinalis*, and *Polygonum hydropiper*. In order to restore the damaged karst ecosystem, based on the previous practice of the research group, artificial grassland was planted in the study area in 2012 by selecting artificial forage that would be suitable for the climatic environment of the region and would produce better ecological and economic benefits. The established forage consists mainly of *Lolium perenne*, *Trifolium repens*, *Dactylis glomerata*, etc. The variety of *Trifolium repens* is “Haifa”, the variety of *Dactylis glomerata* is “Qiangrass No. 4”, and the variety of *Lolium perenne* is “Yaqing”; the three kinds of forage seeds were provided by Guizhou Shennong Seed Industry Co., Ltd. (Guiyang, China) and Lvyi Seed Industry Co., Ltd. (Guiyang, China) (Song et al., 2022). The established artificial grassland has both single and mixed seeding. The seeding rates for *Trifolium repens*, *Dactylis glomerata*, and *Lolium perenne* have been found to be 2.0 g/m^2^, 3.0 g/m^2^, and 3.0g/m^2^, respectively. A base fertiliser (N-P_2_O_5_-K_2_O) was applied before planting at 0.225 g/m^2^. In addition, naturally restored grassland was also present. Therefore, the overall grassland ecosystem formed in the process of karst desertification control includes the natural grassland ecosystem and the artificial grassland ecosystem.

### Sample plot design, measurement of plant functional traits, and field sampling

*Dactylis glomerata* monoculture, *Lolium perenne* monoculture, and a mixture of *Dactylis glomerata* + *Trifolium repens* are common establishment practices in the study area, so three grassland types with similar site conditions were selected as test plots: a *Dactylis glomerata* grassland plot (DG), a *Lolium perenne* grassland plot (LP), and a *Dactylis glomerata* + *Trifolium repens* mixed seeding grassland plot (LT). A natural grassland plot (NG) was selected as a control (**Table 1**). DG, LP, and LT were mowed four times a year and fertilized with N-P_2_O_5_-K_2_O at the time of the first noticeable rainfall after the first and the third mowings, at a rate of 0.1125 g/m^2^. No agricultural activities or human disturbances were carried out at NG. Six sampling plots measuring 10m × 10m were set up for each experimental plot, resulting in a total of 24 sampling plots. Due to the fragmented nature of the surface of the study area, sampling plots were spaced more than 10 meters apart. Five 1m × 1m quadrats were set up in each sampling plot for acquisition of the vegetation community and biomass. The quadrat locations within the sampling plots were chosen randomly, but the locations of each quadrat were 2 m away from each other to avoid edge effects. Vegetation survey and sampling were conducted in mid-August 2021, at the peak of biomass and species diversity, and the names of the species occurring within each quadrat and basic information on them, such as height, coverage, and density, were recorded to assess the richness and diversity of each plant community. The above-ground parts of each species in each quadrat were mowed flush with the ground in their entirety to form a mixed sample, and after removal of any dead parts (litter) adhering to the soil, stones, and other impurities, this sample was oven-dried at 75°C for 48 hours to a constant weight, to be used for calculation of the aboveground biomass.

**Table 1:** Geographical characteristics of sample plots in the study area.

Treatment	Longitude	Latitude	Altitude/m	Slope/°	Coverage/%	Dominant species
NG	105°6′6″E	27°14′49″N	1878	24	82	*Artemisia lavandulaefolia*
DG	105°6′5″E	27°14′26″N	1829	32	84	*Dactylis glomerata*
LP	105°6′6″E	27°14′35″N	1854	21	87	*Lolium perenne*
LT	105°5′59″E	27°14′49″N	1828	20	94	*Lolium perenne*, *Trifolium repens*

DG, *Dactylis glomerata* grassland ecosystem; LP, *Lolium perenne* grassland ecosystem; LT, *Lolium perenne*+*Trifolium repens* grassland ecosystem; NG, natural grassland ecosystem.

Based on the basic characteristics of the sample plots and the results of a previous study (Brown and Anand, 2022), we selected five traits that are closely related to plant life strategies and functional trade-offs: plant height (H, cm), specific leaf area (SLA, cm^2^.g^-1^), leaf dry matter content (LDMC, g·g^-1^), root length (RL, cm), and root dry matter content (RDMC, g·g^-1^). In each quadrat, 3–5 dominant species were randomly selected for measurement of plant height. Leaf samples from healthy and mature plants were taken, in four directions (east, south, west, and north) from each plant, using scissors; approximately 12 leaves were randomly selected for measurement of leaf area; five small sample plots of leaves within each sampling plot were completely and uniformly mixed to form a mixed sample, and young leaves, old leaves, and leaves affected by diseases and insect pests were removed from the sampling process. After removal of the litter layer from the soil surface, soil and root samples were taken from the surface layer (0–20cm) using a soil auger and a root auger, respectively, at the same location. Five soil and root samples from each quadrat were mixed to form a composite sample. If the soil depth was less than 20 cm, samples were taken from the full depth. In total, 24 samples each of leaves, soil, and roots were taken back to the laboratory. The root samples were taken out of the root auger and the root was thoroughly washed; 3–5 root systems were then randomly selected for measurement of the root length with a tape measure. The collected leaves and roots were placed in water and stored in a dark environment at 5°C for 12 h. After the leaves and roots were removed from the water, the water on the surface of the leaves and roots was immediately absorbed with absorbent paper, and the saturated fresh weight of the leaves and roots was obtained by weighing them on an electronic balance at 1/10,000. The dry weight of the fully mixed leaf and root samples was measured by drying at 75°C for 48 h to constant weight; this measurement was used to calculate the dry matter content of the leaves and roots (belowground biomass), respectively. The collected soil samples were divided into two parts: one part was used for the determination of soil field capacity and soil porosity, and the other was used for the determination of soil nutrients. The samples used for the determination of soil nutrients were naturally air-dried indoors (Li et al., 2020). Oven-dried plant samples (leaves and roots) and air-dried soil samples were sieved through 2 mm sieves for the determination of plant and soil nutrient properties, respectively.

Leaf area was measured using a leaf area meter (LI-COR, 3100C Area Meter, USA). SLA is the ratio of leaf area to leaf dry weight; LDMC is the ratio of leaf dry weight to leaf saturated fresh weight; and RDMC is the ratio of root dry weight to root saturated fresh weight. Soil field capacity and soil porosity were determined by the oven-drying method. The potassium dichromate–external heating method was used to determine the organic carbon content of plants and soil; the sulfuric acid catalyst digestion–Kjeldahl method was used to determine the total nitrogen (TN) content of soil; the concentrated sulfuric acid digestion–Mo-Sb colorimetric method was used to determine the total phosphorus (TP) content of soil; and the flame photometric method was used to determine the total potassium (TK) content of soil (Zhang, 2011). TN was determined using a continuous flow analyzer (FLOWSYS, SYSTEA, Italy). TP was determined with an ultraviolet-visible spectrophotometer (Specord 200 PLUS, Analytik, Germany). Finally, TK was determined using an atomic absorption spectrometer (ICE3500, Thermo Fisher, USA).

### Ecosystem function

In accordance with the MEA description of ecosystem function (MEA, 2005) and the ecological restoration goal of control of karst desertification, we selected indicators of grassland ecosystem function such as ecosystem productivity, carbon storage, water conservation, and soil conservation for analysis in this study. Ecosystem productivity was expressed as aboveground biomass and belowground biomass (Wu et al., 2017). Carbon storage was expressed as aboveground plant carbon content, soil carbon content, and root carbon content (Wu et al., 2017). Water conservation was expressed as soil field capacity and soil porosity (Fan et al., 2019). Finally, soil fertility conservation was expressed as TN, TP, and TK (Wu et al., 2017).

### Calculation of indices and data analysis

We used the Margalef index, the Shannon–Wiener index, the Pielou index, and the Simpson index to assess species richness and diversity in the grassland ecosystems. The Margalef index (R), Shannon–Wiener index (H), Pielou index (E), and Simpson index (C) were calculated for each ecosystem using the following formulae:


(Eq. 1) R=(S−1)/InN

(Eq. 2) $$\text{H}=-{\sum_{i=1}^{s}P_{i}\ln P}_{i}$$

(Eq. 3) $$P_{i}=N_{i}/N$$

(Eq. 4) E = H/lnS

(Eq. 5), $$\text{C}=1-\sum_{i=1}^{S}\frac{N_{i}\left(N_{i}-1\right)}{N\left(N-1\right)}$$

where S is the total number of species in the community, $P_{i}\;$ is the frequency of species i in the quadrat, $N_{i}\;$ is the number of individuals of species i in the quadrat, and N is the total number of individuals of all species in the quadrat.

Plant functional traits in the grassland ecosystems were analyzed on the basis of CWM and functional trait diversity. The CWM for each trait in each sample was calculated based on the trait value for the species and the relative abundance of the species or aboveground biomass as weights (Garnier et al., 2004). The calculation formula was as follows:


(Eq. 6) $$\text{CWM}=\sum_{\text{i}=1}^{\text{s}}P_{i}\times X_{i},$$

where S is the total number of species in the community, $P_{i}$ is the aboveground biomass or relative abundance of species i in the quadrat, and $X_{i}$ is the trait value of the species i in the quadrat.

The functional richness index (FRic) indicates the degree of species use of the ecological space; the functional dispersion index (FDis) indicates the degree of spatial dispersion of plant traits; and Rao’s quadratic entropy index (RaoQ) integrates information on species richness and differences in functional characteristics between species (Casanoves et al., 2011). These three indices were chosen to characterize plant functional diversity in this study. They were all calculated in R using the FD package (Mammola et al., 2021).

In order to better compare grassland ecosystem functions and assess the impact of different restoration models, we use the comprehensive index of ecosystem function to evaluate this, following the research method of Kearney et al. (2019). First, the data for each ecosystem function index were standardized to fall within a range between 0.1 and 1.


(Eq. 7) $$\text{Positive indexes}:\;{\text{X}}_{\;i}^{\prime }=0.1+\left(\frac{{\text{X}}_{i}-\text{min}(X_{i})}{\max\left(X_{i}\right)-\min\left(X_{i}\right)}\right)*0.9$$

(Eq. 8) $$\text{Negative indexes}:\;X_{\;i}^{\prime }=1.1-0.1+\left(\frac{{\text{X}}_{i}-\text{min}(X_{i})}{\max\left(X_{i}\right)-\min\left(X_{i}\right)}\right)*0.9,$$

where ${\text{X}}_{\;i}^{\prime }$ is the change value of evaluation index  i , ${\text{X}}_{i}\;$ is the original observation value of evaluation index i , and $\max\left(X_{i}\right)\;$ and $\min\left(X_{i}\right)$ are the maximum observation value and minimum observation value of evaluation index i , respectively.

The comprehensive index of grassland ecosystem function is calculated as the weighted sum of all transformation variables in each group. The weights are determined based on the relative contribution of each variable to the variance within the ecosystem function group using principal component analysis (PCA). The formula used for this calculation was as follows:


(Eq. 9) $$CI=\sum_{i}^{n}\left({\text{X}}_{\;i}^{\prime }Y_{i,PC1}+{\text{X}}_{\;i}^{\prime }Y_{i,PC2}\right),$$

where CI is the comprehensive index of ecosystem function, ${\text{X}}_{\;i}^{\prime }$ is the value converted from Equations 7 or 8 for each evaluation index i (containing n variables), and $Y_{i}$ represents the factor scores on the first and second principal component axes. Finally, each composite index of ecosystem function was again adjusted to a range of 0.1–1 using Equation 7.

One-way analysis of variance (ANOVA) and Tukey pairwise comparisons were performed using IBM SPSS Statistics (version 19.0 for Windows; SPSS, Chicago, IL, USA); these tests were used to analyze the effects of different grassland restoration models on species diversity, CWM, functional trait diversity, and ecosystem function. Tukey pairwise comparisons were considered statistically significant at *P*<0.05. Pearson correlation analysis was conducted to assess the relationships among species diversity, CWM, and functional trait diversity using Origin 2021(version 2021 for Windows; OriginLab, MAS, Hampton, USA). Redundancy analysis (RDA) based on forward selection was carried out to evaluate the effects of species diversity and plant functional traits on ecosystem function using Canoco (version 5.0 for Windows; Ithaca, NY, USA), and the Monte Carlo permutation test was performed to select explanatory factors that had significant effects on changes in ecosystem function (*P<*0.05).

## Results

### Characteristics of species diversity and plant functional traits in grassland ecosystems under different restoration models

We conducted ANOVA and Tukey pairwise comparisons to analyze species diversity, CWM, and functional trait diversity in grassland ecosystems under different restoration models. In terms of species diversity (**Table 2**), the Margalef index and the Shannon–Wiener index were significantly lower in DG and LP than in NG (*P*<0.05), while the Simpson index was significantly higher in the former two ecosystems than in NG (*P*<0.05), but there was no significant difference between DG and LP (*P*>0.05). In addition, there were no significant differences (*P>*0.05) between NG and LT in terms of the Margalef index, Shannon–Wiener index, Pielou index, or Simpson index. Furthermore, the differences in Pielou index between treatments were also not significant (*P>*0.05).

**Table 2:** Species diversity of grassland ecosystems under different restoration models.

Treatment	Margalef index	Shannon–Wiener index	Pielou index	Simpson index
NG	1.08 ± 0.17a	1.10 ± 0.19a	0.63 ± 0.12a	0.47 ± 0.12b
DG	0.68 ± 0.21b	0.71 ± 0.20b	0.51 ± 0.07a	0.64 ± 0.10a
LP	0.55 ± 0.12bc	0.67 ± 0.12bc	0.54 ± 0.07a	0.64 ± 0.05a
LT	1.19 ± 0.34a	1.16 ± 0.10a	0.63 ± 0.07a	0.46 ± 0.04bc
*F* value	11.143**	15.11**	3.075	9.451**

Data are presented in the form mean ± standard error. Different letters in the same column indicate significant differences (*p*<0.05) between treatments based on Tukey pairwise comparisons. ** indicates significance at the 0.01 probability level. The *F* value is the F test statistic.

DG, *Dactylis glomerata* grassland ecosystem; LP, *Lolium perenne* grassland ecosystem; LT, *Lolium perenne* + *Trifolium repens* grassland ecosystem; NG, natural grassland ecosystem.

**Figure 1:**
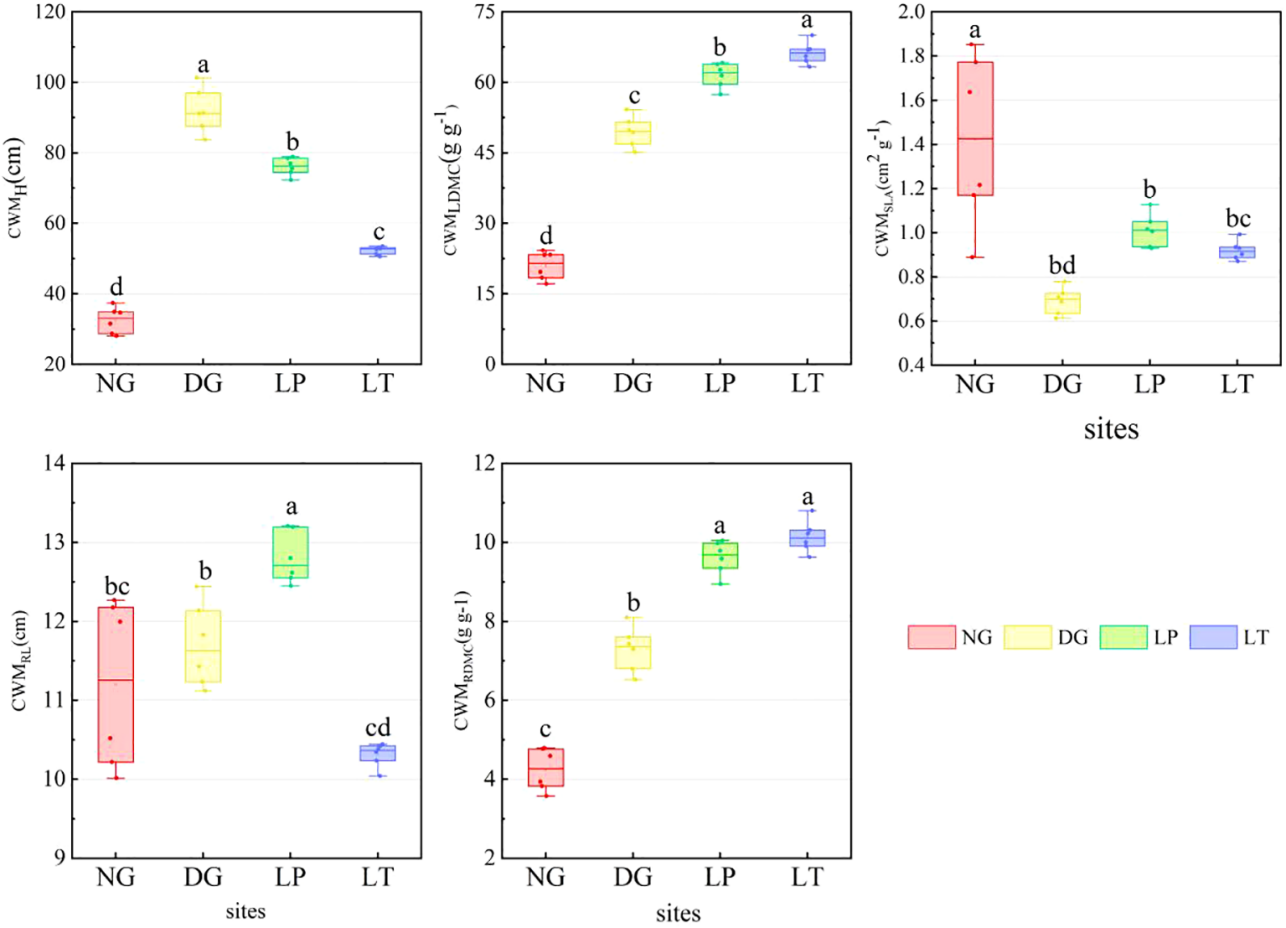
CWM of grassland ecosystems under different restoration models. Different letters in the same panel indicate significant differences between treatments based on Tukey pairwise comparisons (*p*<0.05). CWM_H_, CWM_LDMC_, CWM_SLA_, CWM_RL_, and CWM_RDMC_ represent the CWM for plant height, leaf dry matter content, specific leaf area, root length, and root dry matter content, respectively.DG, *Dactylis glomerata* grassland ecosystem; LP, *Lolium perenne* grassland ecosystem; LT, *Lolium perenne + Trifolium repens* grassland ecosystem; NG, natural grassland ecosystem.

The CWM of grassland ecosystems under different restoration models varied significantly (**Figure 1**). CWM_H_ ranged from 28.02 to 101.30 cm. The distribution of CWM_H_ was more concentrated in LT, while the variance of CWM_H_ was greatest in DG. Compared to NG, CWM_H_ was significantly higher in DG, LP, and LT (*P*<0.05). The median variation in CWM_LDMC_ ranged from 21.49 to 66.28 g·g^-1^. CWM_LDMC_ was highest in LT and lowest in NG. CWM_SLA_ varied from 0.61 to 1.85 cm^2^·g^-1^. The distribution of CWM_SLA_ was more concentrated in LT, while the variance of CWM_SLA_ was greatest in NG. CWM_SLA_ was significantly higher in NG than in other grassland ecosystems (*P*<0.05), but there were no significant differences among DG, LP, and LT (*P*>0.05). The median variation in CWM_RL_ ranged from 10.37 to 12.71 cm. CWM_RL_ was highest in LP (*P*<0.05), but there was no significant difference in CWM_RL_ between NG and DG, or between DG and LT (*P*>0.05). CWM_RDMC_ ranged from 3.58 to 10.80 g·g-1. CWM_RDMC_ was significantly higher in DG, LP, and LT than in NG (*P*<0.05), but there was no significant difference between LT and LP (*P*>0.05).

**Figure 2:**
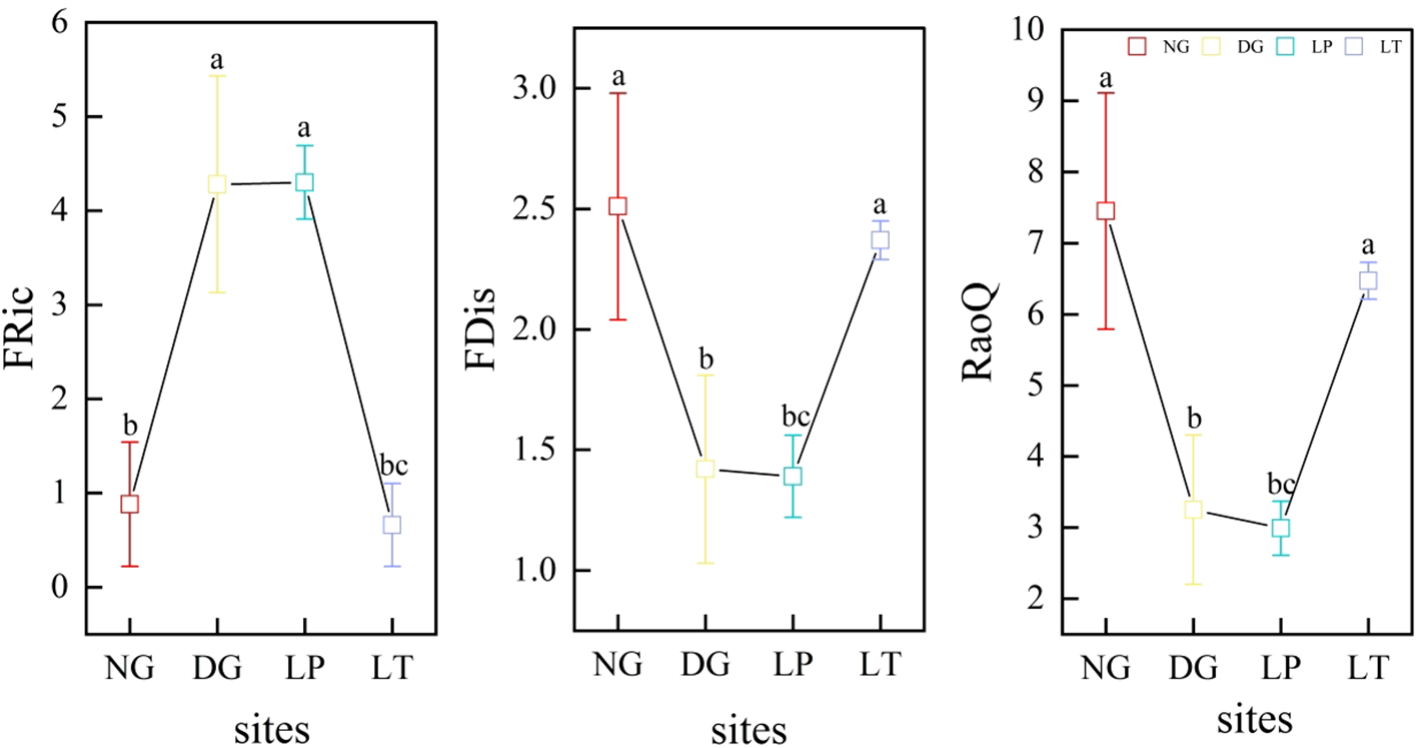
Functional trait diversity of grassland ecosystems under different restoration models. Different letters in the same panel indicate significant differences between treatments based on Tukey pairwise comparisons (*p*<0.05). FRic, FDis, and RaoQ represent the functional richness index, the functional dispersion index, and Rao’s quadratic entropy index, respectively. DG, *Dactylis glomerata* grassland ecosystem; LP, *Lolium perenne* grassland ecosystem; LT, *Lolium perenne + Trifolium repens* grassland ecosystem; NG, natural grassland ecosystem.

It was not difficult to observe that functional trait diversity in grassland ecosystems varied greatly among different restoration models (**Figure 2**). FRic was significantly higher in DG and LP than in NG and LT (*P*<0.05), but there was no significant difference between DG and LP, and there was also no significant difference between NG and LT (*P*>0.05). FDis and RaoQ were significantly lower in DG and LP than in NG (*P*<0.05), but there was no significant difference between DG and LP (*P*>0.05), and there was also no significant difference between NG and LT (*P*>0.05).

**Figure 3:**
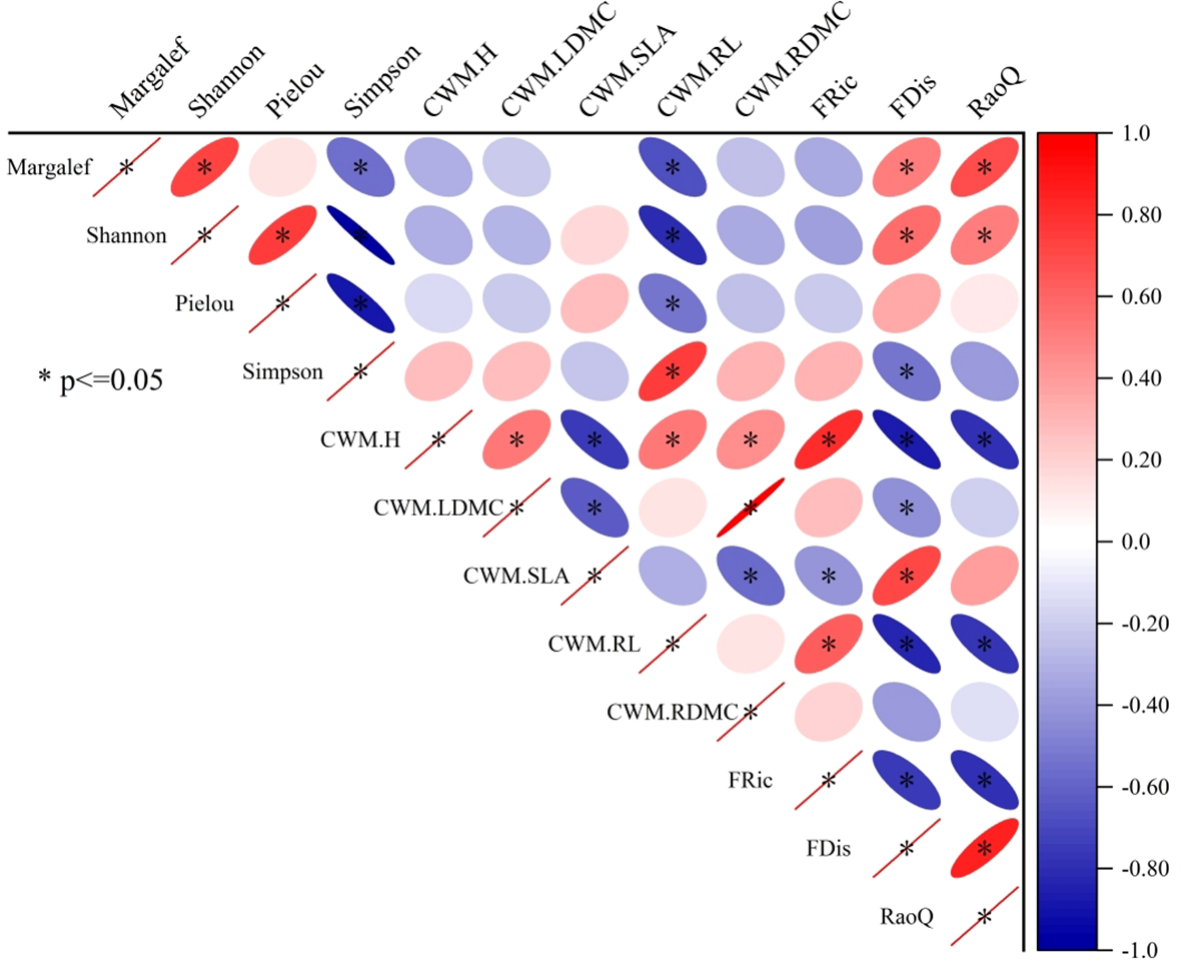
Correlation analyses of species diversity, CWM, and functional trait diversity in grassland ecosystems under different restoration models. * indicates significance at the 0.05 probability level. The labels Margalef, Shannon, Pielou, and Simpson represent the Margalef index, the Shannon–Wiener index, the Pielou index, and the Simpson index, respectively. CWM.H, CWM.LDMC, CWM.SLA, CWM.RL, and CWM.RDMC represent the CWM of plant height, leaf dry matter content, specific leaf area, root length, and root dry matter content, respectively. FRic, FDis, and RaoQ represent the functional richness index, the functional dispersion index, and Rao’s quadratic entropy index, respectively.

We performed correlation analyses of species diversity, CWM, and functional trait diversity in grassland ecosystems under different restoration models (**Figure 3**). Margalef index was positively correlated with Shannon–Wiener index, FDis, and RaoQ, but negatively correlated with Simpson index and CWM_RL_ (*P*<0.05). Shannon–Wiener index was positively correlated with Pielou index, FDis, and RaoQ (*P*<0.05). Shannon–Wiener index and Pielou index were negatively correlated with Simpson index and CWM_RL_ (*P*<0.05). Simpson index was positively correlated with CWM_RL_ (*P*<0.05), but negatively correlated with FDis (*P*<0.05). CWM_H_ was positively correlated with CWM_LDMC_, CWM_RL_, CWM_RDMC_, and FRic, but negatively correlated with CWM_SLA_, FDis, and RaoQ (*P*<0.05). CWM_LDMC_ was positively correlated with CWM_RDMC_, but negatively correlated with CWM_SLA_ and FDis (*P*<0.05). CWM_SLA_ was positively correlated with FDis but negatively correlated with CWM_RDMC_ and FRic (*P*<0.05). CWM_RL_ was positively correlated with FRic but negatively correlated with FDis and RaoQ (*P*<0.05). FRic was negatively correlated with FDis and RaoQ (*P*<0.05). Finally, FDis was positively correlated with RaoQ (*P*<0.05).

### Characteristics of grassland ecosystem function under different restoration models

We used ANOVA and Tukey pairwise comparisons to analyze indices of grassland ecosystem function under different restoration models (**Table 3**). Aboveground biomass was greatest in LT, but there was no significant difference between DG and LP (*P*>0.05), and there was also no significant difference between DG and NG (*P*>0.05). Compared to NG, the aboveground biomass in DG, LP, and LT was greater by approximately 20%, 30%, and 44%, respectively. Belowground biomass was greatest in LT and lowest in NG, but there was no significant difference between DG and LP (*P*>0.05). Compared to NG, the belowground biomass in DG, LP, and LT was greater by approximately 32%, 35%, and 56%, respectively. Plant carbon content was significantly higher in DG, LP, and LT than in NG (*P*<0.05), but there were no significant differences among DG, LP, and LT (*P*>0.05). Carbon content in the roots was lowest in NG, but there was no significant difference between DG and LP, and there was also no significant difference between DG and LT (*P*>0.05). Soil organic carbon, soil field capacity, soil porosity, TN, TP, and TK were significantly higher in LT than under the other treatments (*P*<0.05). These indices were lowest in NG, but there was no significant difference between DG and LP (*P*>0.05), except in the case of TP.

**Table 3:** Analysis results for each evaluation index for grassland ecosystem functions under different restoration models.

EF group	NG	DG	LP	LT	*F*
Ecosystem productivity					
Aboveground biomass (dry weight, kg·m^2^)	0.28 ± 0.03dc	0.35 ± 0.03bc	0.40 ± 0.05b	0.50 ± 0.04a	28.01***
Belowground biomass (dry weight, kg·m^2^)	0.15 ± 0.01d	0.22 ± 0.01bc	0.23 ± 0.01b	0.34 ± 0.02a	237.51***
Carbon storage					
Plant C content (g·kg^-1^)	406.61 ± 9.37b	461.11 ± 8.64a	456.15 ± 24.69a	481.14 ± 33.26a	12.81***
Root C content (g·kg^-1^)	321.43 ± 11.08c	368.63 ± 17.93ab	350.44 ± 19.45b	380.91 ± 11.65a	16.73***
Soil organic carbon (g·kg^-1^)	12.51 ± 2.19d	17.62 ± 2.37bc	18.52 ± 3.11b	25.32 ± 3.11a	32.31***
Water conservation					
Soil field capacity (%)	27.97 ± 5.02d	36.31 ± 2.82bc	36.55 ± 1.66b	43.09 ± 2.29a	22.33***
Soil porosity (%)	41.78 ± 1.32d	47.88 ± 2.42bc	48.59 ± 0.67b	53.92 ± 3.30a	31.32***
Soil fertility conservation					
Total nitrogen (g·kg^-1^)	0.79 ± 0.05d	1.53 ± 0.24bc	1.66 ± 0.46b	2.14 ± 0.28a	21.63***
Total phosphorus (g·kg^-1^)	0.81 ± 0.05cd	0.95 ± 0.03c	1.11 ± 0.13b	1.38 ± 0.11a	44.60***
Total potassium (g·kg^-1^)	15.06 ± 0.91d	16.82 ± 0.39bc	17.23 ± 0.71b	22.21 ± 1.09a	84.73***

Data are presented in the form mean ± standard error. Different letters in the same column indicate significant differences (*p*<0.05) between treatments based on Tukey pairwise comparisons. *** indicates significance at the 0.001 probability level. The *F* value is the F test statistic.

DG, *Dactylis glomerata* grassland ecosystem; LP, *Lolium perenne* grassland ecosystem; LT, *Lolium perenne* + *Trifolium repens* grassland ecosystem; NG, natural grassland ecosystem.

**Figure 4:**
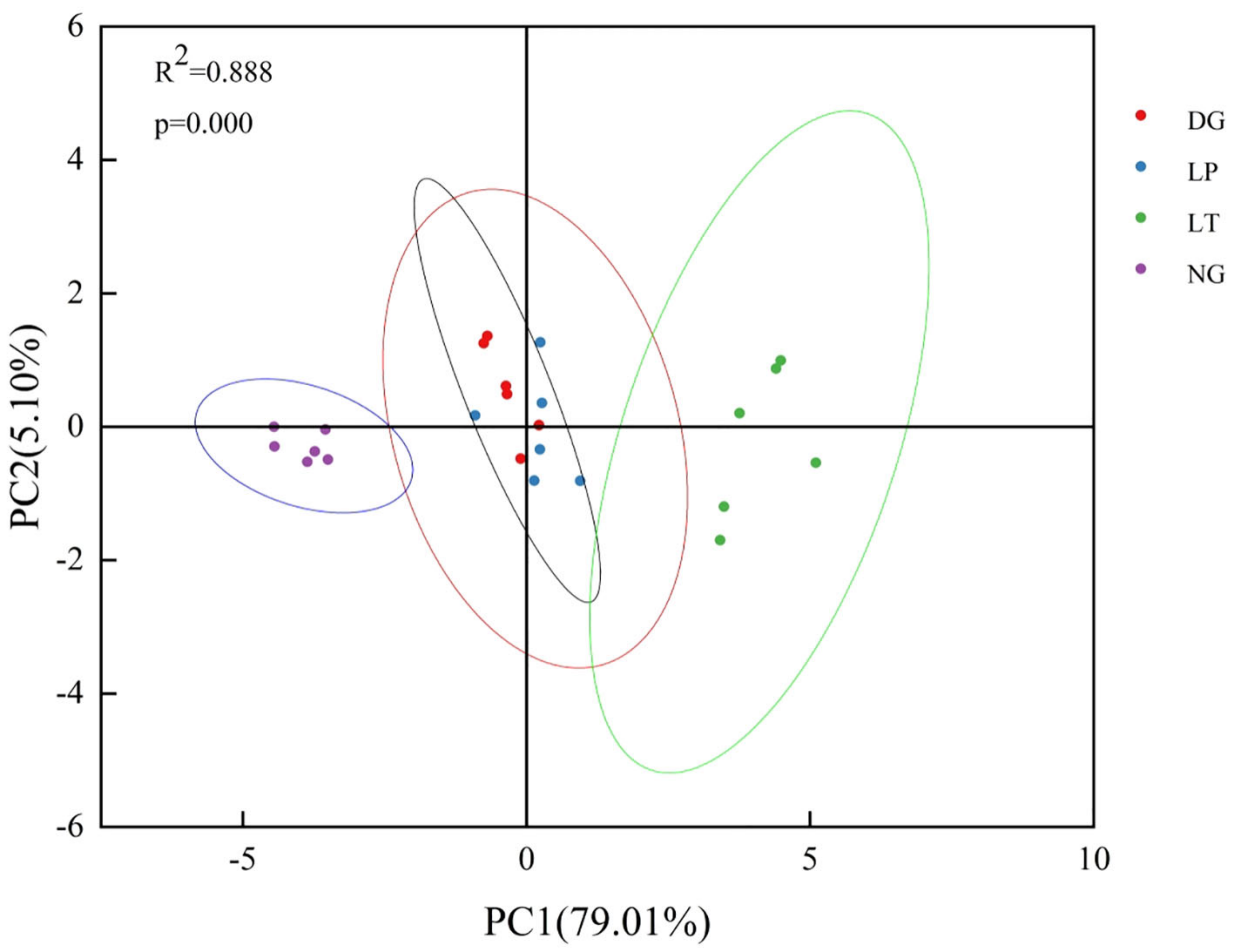
Principal component analysis of each indicator for evaluation of grassland ecosystem function under different restoration models.

The results of PCA showed that the first and second principal components together explained 84.11% of the variance in each ecosystem function group for each evaluation indicator (**Figure 4**). Based on the explanatory power of the principal component and using this as a weight to calculate the comprehensive index of ecosystem function via Equation 9, we obtained values for the comprehensive index of ecosystem function for grassland ecosystems under different restoration models (**Table 4**). The same trends in terms of differences between restoration models were observed across multiple components of the comprehensive index of grassland ecosystem function: that is, ecosystem productivity, carbon storage, water conservation, and soil fertility were highest in LT and lowest in NG, and there was no significant difference between DG and LP (*P*>0.05).

**Table 4:** Comprehensive index of grassland ecosystem function under different restoration models.

Comprehensive index of EF	NG	DG	LP	LT	*F*
**Ecosystem productivity**	0.21 ± 0.05d	0.46 ± 0.05bc	0.55 ± 0.07b	0.94 ± 0.06a	152.947***
**Carbon storage**	0.25 ± 0.09d	0.64 ± 0.04b	0.58 ± 0.08bc	0.87 ± 0.09a	63.356***
**Water conservation**	0.18 ± 0.09d	0.53 ± 0.13bc	0.56 ± 0.04b	0.86 ± 0.01a	48.161***
**Soil fertility conservation**	0.16 ± 0.04d	0.42 ± 0.06bc	0.53 ± 0.12b	0.90 ± 0.07a	102.52***

Data are presented in the form mean ± standard error. Different letters in the same column indicate significant differences (*p*<0.05) between treatments based on Tukey pairwise comparisons. The *F* value is the F test statistic. *** indicates significance at the 0.001 probability level.

DG, *Dactylis glomerata* grassland ecosystem; LP, *Lolium perenne* grassland ecosystem; LT, *Lolium perenne* + *Trifolium repens* grassland ecosystem; NG, natural grassland ecosystem.

### Relationship between plant functional traits and grassland ecosystem function under different restoration models

**Figure 5:**
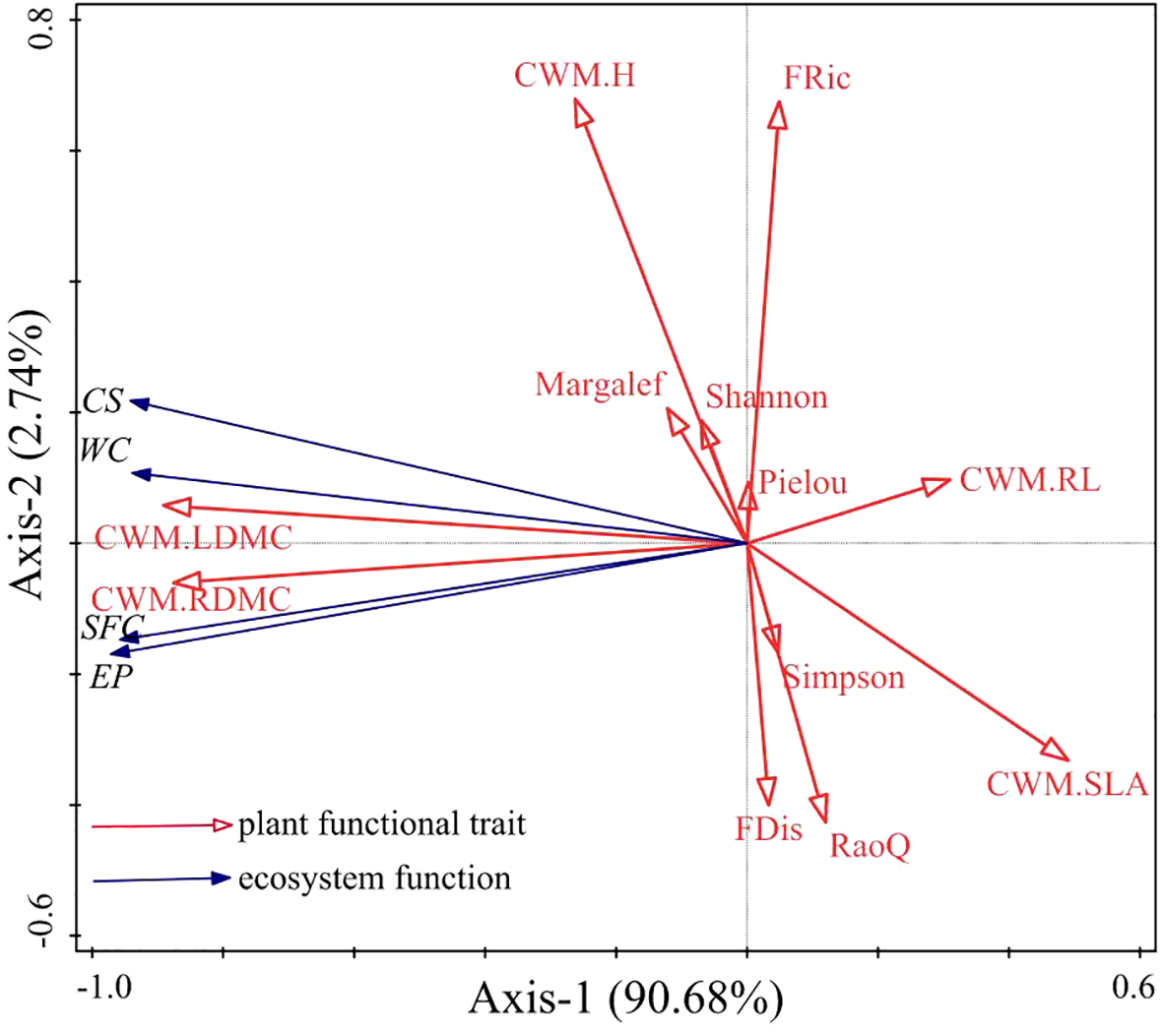
Redundancy analysis of plant functional traits and grassland ecosystem function. The labels Margalef, Shannon, Pielou, and Simpson represent the Margalef index, the Shannon–Wiener index, the Pielou index, and the Simpson index, respectively. CWM.H, CWM.LDMC, CWM.SLA, CWM.RL, and CWM.RDMC represent the CWM of plant height, leaf dry matter content, specific leaf area, root length, and root dry matter content, respectively. FRic, FDis, and RaoQ represent the functional richness index, the functional dispersion index, and Rao’s quadratic entropy index, respectively. EP, CS, WC, and SFC represent ecosystem productivity, carbon storage, water conservation, and soil fertility conservation, respectively.

Redundancy analysis of plant functional traits and ecosystem function showed that the first and second axes explained 90.68% and 2.74% of the variance in ecosystem function, respectively (**Figure 5**). CWM_LDMC_, CWM_RDMC_, CWM_H_, the Margalef index, and the Shannon-Wiener index were positively correlated with ecosystem productivity, carbon storage, water conservation, and soil fertility conservation. CWM_SLA_ and CWM_RL_ were negatively correlated with ecosystem productivity, carbon storage, water conservation, and soil fertility conservation. FRic, FDis, and RaoQ were not correlated with ecosystem productivity, carbon storage, water conservation, or soil fertility conservation. Further analysis via the Monte Carlo permutation test showed that CWM_LDMC_, CWM_RL_, and CWM_H_ had larger effects on ecosystem function than other plant functional traits, explaining 76.20% (F=56.7, P=0.024), 17.7% (F=28.7, P=0.024), and 2.2% (F=4.5, P=0.048) of the variance, respectively, indicating that CWM_LDMC_, CWM_RL_, and CWM_H_ were the main factors affecting ecosystem function.

## Discussion

### Effects of different restoration models on species diversity and plant functional traits in grassland ecosystems

Species diversity represents the expression of biodiversity at the species level. As a fundamental characteristic of community functional structure, this measure plays a crucial role in indicating community composition, the dynamics of community change, and strategies for vegetation restoration (Crawley and Harral, 2001). Under various restoration models, variation has been observed in species composition and structure among plant communities, resulting in significant disparities in species diversity (Wang et al., 2022a). In this study, plant species diversity under the three artificial restoration models varied greatly. The Margalef index and Shannon index were highest in LT, and the species diversity of the LT ecosystem was closest to that of NG, which is consistent with the findings of Wu et al. (2019) in the degraded grassland of the Three Rivers source area and those of Zhang H. et al. (2020) in the degraded grassland of the Yellow River headwaters. This indicates that, for degraded ecosystems, planting artificial grassland by mixed sowing can promote the vegetation succession of plant communities and restore species diversity in the degraded ecosystem, which is an important way to restore degraded ecosystems. However, there was no significant difference in Margalef index, Shannon–Wiener index, or Pielou index between the DG and LP ecosystems; this finding was similar to the results of Hou et al. (2015) in the *Poa pratensis* grassland of Qinghai, but different from the results of Hao et al. (2023) in the sandy grassland of Hulunbeir. This phenomenon could potentially be attributed to the similarity in dominant species between DG and LP, as well as the nature of the study conducted by Hou et al. (2015), in which both areas featured perennial grasses. Perennial grasses share commonalities in terms of morphology, physiology, and life history, which may explain the observed similarities.

Plant functional traits determine plant survival, growth, and reproduction; not only can these characteristics strongly influence ecosystem processes, but they also reflect the response process of ecosystems to environmental changes (Wright et al., 2004; Kraft et al., 2015; Li et al., 2021). The adaptability of plants to the environment and their own plasticity leads to major differences in functional traits among plants, with species themselves often acting as indicators and predictors of environmental change through their adoption of morphological and physiological traits to adapt to environmental changes and access to limited resources (Freschet et al., 2018; Hu L. et al., 2021). We found that the five CWM measures responded very differently to different restoration models during the ecosystem restoration process for management of karst desertification. CWM_H_, CWM_LDMC_, and CWM_RDMC_ were significantly higher in artificial grassland ecosystems than in the natural grassland ecosystem, which can be explained by the fact that artificial planting promotes forage with rapid growth strategies as dominant species, and that local dominance of relatively tall plants can lead to higher plant biomass and higher resource storage capacity (Conti and Díaz, 2013; Zuo et al., 2017). However, the CWM_SLA_ of plants in artificial grassland ecosystems was lower, which may be due to the fact that the karst ecosystem in southwest China has specific habitat conditions consisting of karst aridity and high soil calcium, and the plants have acquired resilience to this by altering their own structure and their physiological and biochemical processes to adapt to the environment in the course of survival (Xiong et al., 2002; Xi et al., 2011; Zhong et al., 2018). While SLA is related to stress tolerance and growth rate, species with lower SLA can compete for limited nutrients and have higher stress tolerance (Wright et al., 2004), which also proves that artificially planted grass is better adapted to the particular nature of the karst environment. This result was similar to those of Mason et al. (2016) in grazed grassland in New Zealand. Plants obtain nutrients and water from the soil, mainly through the root system, to support their growth and development, and the root system is the area where the most intense plant–soil interactions take place, largely controlling nutrient activation, water use efficiency, and soil health (Kong et al., 2014; Plaza-Bonilla et al., 2014). In this study, CWM_RL_ was significantly higher in DG and LP than in LT, which is consistent with the results of Li et al. (2022) in grassland in Inner Mongolia. This may be attributable to the fact that gramineous grasses have well-developed fibrous roots with a strong ability to compete for water, and water competition intensifies the growth of gramineous grasses roots in monoculture (Bessler et al., 2012).

The concept of functional trait diversity is intricately linked to the functions of species and ecosystems, as it involves the complementary utilization of resources (Mason et al., 2005). This approach provides a clear and intuitive means of characterizing the magnitude, distribution range, and degree of niche differentiation of functional traits within an ecosystem community (Villéger et al., 2008). The FRic reflects the utilization rate of resources by vegetation by quantifying the niche space occupied by existing species in the community (Jäschke et al., 2020). This study showed that FRic was significantly higher in DG and LP than in NG and LT, indicating that the dominant species of perennial gramineous grasses in the community occupied a larger niche space, the niche differentiation of each species was higher, and resource utilization was more efficient (Petchey and Gaston, 2006; Garcia-Cervigon et al., 2021). This result is inconsistent with the findings of Perring et al. (2018) in European temperate grassland and Freitag et al. (2021) in the Eurasian steppe, which may be caused by the particular nature of the habitat environment of karst (Huang et al., 2022). FDis and RaoQ can be used to measure the degree of niche differentiation and resource competition among plants within a community (Carmona et al., 2016), with higher index values indicating stronger ecological niche complementarity among species, weaker competition, and more efficient resource use (Dong et al., 2019). In this study, FDis and RaoQ were significantly higher in NG and LT than in DG and LP, which is consistent with the results of a related study by Huang et al. (2020) in Chongqing City in China. This may be due to the fact that natural and mixed-seeding grassland ecosystems have a great variety and number of species, producing differences in niches between species, and leading to less niche overlap in resource utilization by individuals in natural and mixed-seeding grassland ecosystems compared to single-seeding grassland ecosystems (Yang et al., 2022).

In this study, the Margalef index and Shannon index were higher in NG and LT than in DG and LP, but FRic did not show the same trend. This may be due to the fact that, in degraded karst ecosystems where species are under environmental stress, species trait show convergence in their functional traits, species trait composition is limited to traits adapted to the selective pressures of that environment, and continued increases in species richness only lead to further spatial differentiation of ecological niches, resulting in reduced interspecific trait variability and no further increase in functional diversity (Sasaki et al., 2009; Khasanova et al., 2019), as well as weakened competition between species. These findings are consistent with the results of Dong et al. (2019) on the adaptive strategies of plants in grassland ecosystems of the Tibetan Plateau and with those of Zhang Z. et al. (2021) in nine forest ecosystems from the tropical to boreal zones in China. The results of this study confirmed the following findings: 1) FDis and RaoQ were significantly higher in NG and LT compared to DG and LP; 2) the Margalef index and Shannon index were significantly positively correlated with FDis and RaoQ, but not significantly correlated with FRic; 3) CWM_H_ and CWM_RL_ were significantly negatively correlated with FDis and RaoQ. In addition, the relationship between the Margalef index and Shannon index, as well as functional trait diversity, varied across different types of plant communities. This was confirmed by Weng et al. (2017) through their study of forest communities, suggesting that the relationship between species diversity and functional diversity has originally varied across environmental contexts, even when these are unaffected by external disturbance.

In this study, CWM_H_ was significantly and positively correlated with CWM_LDMC_, CWM_RL_, CWM_RDMC_, and FRic. This may be due to the fact that plant height affects the ability of plants to acquire light and to photosynthesize; specifically, the greater a plant’s height, the greater its ability to so, which contributes to an increase in leaf dry matter content, root length, and root dry matter content. In turn, these also contribute to an increase in the diversity of plant functional traits (Angassa, 2014). These results are similar to those obtained by Maire et al. (2015) in their study of global terrestrial ecosystems. SLA is an important indicator for measurement of the growth status and light energy utilization efficiency of species, and LDMC mainly reflects the ability to retain plant nutrient elements (Hao et al., 2019). Related studies have shown that SLA can reflect the ability of plants to obtain resources, and SLA is usually negatively correlated with LDMC (Shen et al., 2019), which may explain the findings of a significant negative correlation between CWM_LDMC_ and CWM_SLA_ in this study. This is consistent with the results of Niu et al. (2016) in Tibetan alpine meadows, those of Zhang et al. (2018) in typical grassland of Horqin sandy land, China, and those of Wang Q. et al. (2022) in degraded woody plants of a karst area. This finding also indicates that the artificial grass in our study area was better adapted to the karst environment, and thus their LDMC was higher. Furthermore, FRic was negatively correlated with FDis and RaoQ, but FDis was positively correlated with RaoQ in our study. This is inconsistent with the results of Jiang X. et al. (2022) in the Mu Us sandy grassland of China and with those of Wang et al. (2022b) in the Maolan National Karst Forest Nature Reserve, Guizhou, China; however, it is consistent with the results of Petchey and Gaston (2002) on five typical cases of simulated plant functional traits and ecosystem relationships, and with those of Hao et al. (2019) on the functional traits of communities at different stages of succession in the temperate forests of the Changbai Mountains, Northeast China. The reason for this difference may be inconsistencies in species diversity.

### Effects of different restoration models on grassland ecosystem function

The aim of ecological restoration is not only to increase vegetation coverage but also to restore ecosystem quality and function (Zhang M. et al., 2018; Zhang S. et al., 2023). Artificial grassland is an effective way to mitigate the degradation of natural grassland, improve grassland productivity, and ensure ecological security (He et al., 2020; Xu et al., 2020). In this study, DG, LP, and LT were found to have significantly increased aboveground biomass and belowground biomass in the grassland ecosystem compared to NG, with the highest levels being observed in LT. These results are consistent with those of Fox et al. (2020) in mixed seeding grassland in Zurich and those of Li C. et al. (2023) in artificial grassland in Qinghai Lake Basin; this may be because artificial pasture planting has some advantages in increasing forage yield compared to natural grassland, but the advantages of mixed seeding are greater than those of monoculture (Husse et al., 2016). The same trend was also observed in plant, root, and soil total carbon, which indicates that the process of accumulation of soil nutrients was slow during the natural process of recovery of the karst ecosystem, while the mixed-seeding grassland could significantly increase soil organic carbon; the effect of mixed leguminous forage has been found to be especially clear (Bai and Cotrufo, 2022). Furthermore, there is a positive correlation between plant and root carbon and soil carbon, with increases in soil carbon promoting higher plant and root carbon (Su et al., 2019). The results of Peng et al. (2020), in a comparative analysis of alpine meadow and alpine steppe, have also confirmed this viewpoint; this may be due to the fact that the soil environment of artificial grassland tends to be stable, with an increase in surface litter and in the underground root system, which is capable of sustainably recharging litter into the soil, increasing the source of soil organic carbon and promoting organic carbon accumulation (Li X. et al., 2023). Soil water is an important source of plant water and a carrier for nutrient cycling and material transformation (Yang et al., 2016). Soil porosity is an important indicator for characterization of soil aeration and water permeability, and soil with high porosity is more likely to expel water (Zhang et al., 2019). Not only can soil organic carbon enhance the soil’s ability to hold and release fertilizer, but it can also promote the formation of granular structure and improve soil’s water permeability, water-holding capacity, and aeration (Zhang J. et al., 2021). This can explain the findings of this study in which soil field capacity and soil porosity were the highest in LT and the lowest in NG, which is consistent with the results of Yang et al. (2017) study of ecological restoration projects affecting hydrological function in the karst region of southwest China.

Soil plays a crucial role in the formation of grassland ecosystem function and the provision of ecosystem services, as it carries out nutrient cycling (Eekeren et al., 2010; Bell et al., 2012). Relevant studies have shown that restoration of vegetation can effectively improve soil fertility within a relatively short period of time (15 years) (Hu et al., 2020). Soil nitrogen supply plays an important role in determining ecosystem structure and function (Hu P. et al., 2021), and the availability of soil nitrogen is often a key limiting factor for productivity (Wang D. et al., 2020). In this study, DG, LP, and LT significantly increased soil total nitrogen content compared to NG, and this was at its highest in LT. This may be due to the relative stability of the mixed-seeding grassland, which enhances photosynthesis, root activity, and soil microbial activity, and promotes nitrogen accumulation capacity; additionally, the biological nitrogen fixation of legume forage in LT also increased nitrogen content, meaning that the presence of mixed-seeding grassland was more conducive to the accumulation of soil total nitrogen (Nyfeler et al., 2011; Frankow-Lindberg and Dahlin, 2013). This is consistent with results on the planting years of different grasslands obtained in the Sanjiangyuan region of China by Xing et al. (2020), but inconsistent with the results of Hu et al. (2021), who studied the effects of vegetation restoration methods on soil N supply in artificial and natural forests in the karst region of southwest China; this might be related to the different vegetation types studied (Song et al., 2022). Soil phosphorus and potassium are the main nutrients affecting plant growth, and higher levels of phosphorus and potassium imply good soil fertility status and higher system productivity (Cheng et al., 2016; Wang et al., 2019; Zhang Y. et al., 2021). In this study, DG, LP, and LT significantly increased the total phosphorus content compared to NG, and this was at its highest in LT. This may be because the biomass, soil organic carbon, and total nitrogen content were significantly higher in artificial grassland than in natural grassland, providing a favorable soil environment for the accumulation of phosphorus and potassium; because mixed-seeding grassland creates inter-root space for the use of phosphorus and potassium nutrients; and because the advantage of the difference in the utilization of phosphorus and potassium sources contributes to a more pronounced increase in soil phosphorus and potassium content (Wagg et al., 2014; Crème et al., 2016). Although the species diversity in the natural grassland ecosystem was higher than that observed in the single-seeding grassland ecosystem in this study, N is a limiting factor affecting the productivity of natural grassland at present (Song et al., 2022). In contrast, the local dominance of high-productivity species in artificial grassland ecosystems promotes the improvement of overall primary productivity (Duchini et al., 2016), and the ecosystem function of artificial grassland is significantly enhanced under plant and soil interaction. Therefore, the interaction of the above factors resulted in the highest ecosystem productivity, carbon storage, water conservation, and soil fertility conservation occurring in LT, with the lowest occurring in NG.

Although the importance of species diversity and plant functional traits in maintaining ecological functions is still debated, numerous studies have shown that both factors have positive effects on ecosystem function (Cadotte, 2017; Armstrong et al., 2021; Chaves et al., 2021; Gao et al., 2022; Dendoncker et al., 2023; Yan et al., 2023). In this study, CWM_LDMC_, CWM_RDMC_, CWM_H_, the Margalef index, and the Shannon index were positively correlated with ecosystem productivity, carbon storage, water conservation, and soil fertility conservation. This indicates that the functioning of the grassland ecosystem in this region is influenced by both species richness and plant functional traits. CWM can be used to assess the effect of dominant traits on ecosystem function, while the functional trait diversity index can quantify the effect of variation in the trait on ecosystem function (Choudhury et al., 2022). The results of RDA in this study showed that the CWM explained the effects of grassland ecosystem function to a greater extent than the functional trait diversity index and the species diversity index, indicating that the dominant species in the community had a greater influence on ecosystem productivity, carbon storage, water conservation, and soil fertility conservation. This suggests that ecosystem function may depend on the functional traits of the dominant species in the community. Specifically, CWM_H_, CWM_LDMC_, and CWM_RL_ were the main factors affecting grassland ecosystem function. This result has also been obtained in studies of degraded grassland ecosystems in the Loess Plateau of China (Jing et al., 2019), Selside meadows in the UK (Sweeney et al., 2020), and subtropical forest ecosystems in the central Himalayas (Sigdel et al., 2022). Therefore, in this study, the mass-ratio hypothesis was found to better explain the response of grassland ecosystem function to plant functional traits under different vegetation restoration models in this region in comparison to the niche differentiation hypothesis.

## Conclusions

This research focuses on the desertification control in the karst ecosystem and examines the impact of plant functional traits on the functioning of grassland ecosystems under various vegetation restoration models in an area of karst desertification. In this study, species diversity (assessed via the Margalef index and Shannon index), FDis, and RaoQ were higher in the grassland ecosystem under the natural restoration model than in ecosystems under artificial restoration models, but the differences between the NG and LT were not significant. Furthermore, grassland ecosystem function, including ecosystem productivity, carbon storage, water conservation, and soil fertility conservation, was found to be at its lowest in NG and highest in LT. This is because the CWM explains a larger proportion of the variance in grassland ecosystem function than the functional trait diversity index, and changes in ecosystem function depend largely on the functional traits of the dominant species in the community. Therefore, we conclude that the mass-ratio hypothesis can better explain the response of grassland ecosystem function to plant functional traits under different vegetation restoration models in this region in comparison to the niche differentiation hypothesis. This may be due to the relatively short monitoring period; in order to study changes in the underlying mechanism, we will need to carry out long-term monitoring. In addition, while plant functional traits have a significant impact on grassland ecosystem function in the area of karst desertification, it is important to consider the effects of climate, soil, and anthropogenic interference on ecosystem recovery in future research. This study proved that, among the different vegetation restoration models employed in the area of karst desertification, ecosystem function was the best in the case of mixed-seeding grassland (perennial ryegrass + white clover), which indicates that degraded karst ecosystem function can be restored by the method of mixed-seeding grassland.

## Data availability statement

The datasets presented in this study can be found in online repositories. The names of the repository/repositories and accession number(s) can be found below: Data available via the figshare https://doi.org/10.6084/m9.figshare.22758158.v1.

## Author contributions

SS and KX performed the sampling and contributed to the experimental design, data analysis, and writing of the manuscript. KX provided the study site and funding. KX and YC contributed to the sampling, experimental design, data analysis, and writing of the manuscript. All authors contributed to the article and approved the submitted version.
